# Infections in Thalassemia and Hemoglobinopathies: Focus on Therapy-Related Complications

**DOI:** 10.4084/MJHID.2009.028

**Published:** 2009-12-28

**Authors:** Bianca Maria Ricerca, Arturo Di Girolamo, Deborah Rund

**Affiliations:** 1 Hematology Department, Catholic University, Rome (Italy); 2 Infectious Diseases Department, G. d’Annunzio University, Chieti-Pescara (Italy); 3 Hebrew University-Hadassah Medical Center, Ein Kerem, Jerusalem, Israel IL 91120

## Abstract

The clinical approach to thalassemia and hemoglobinopathies, specifically Sickle Cell Disease (SCD), based on transfusions, iron chelation and bone marrow transplantation has ameliorated their prognosis. Nevertheless, infections still may cause serious complications in these patients. The susceptibility to infections in thalassemia and SCD arises both from a large spectrum of immunological abnormalities and from exposure to specific infectious agents. Four fundamental issues will be focused upon as central causes of immune dysfunction: the diseases themselves; iron overload, transfusion therapy and the role of the spleen. Thalassemia and SCD differ in their pathogenesis and clinical course. It will be outlined how these differences affect immune dysfunction, the risk of infections and the types of most frequent infections in each disease. Moreover, since transfusions are a fundamental tool for treating these patients, their safety is paramount in reducing the risks of infections. In recent years, careful surveillance worldwide and improvements in laboratory tests reduced greatly transfusion transmitted infections, but the problem is not completely resolved. Finally, selected topics will be discussed regarding Parvovirus B19 and transfusion transmitted infections as well as the prevention of infectious risk postsplenectomy or in presence of functional asplenia.

## Introduction:

Infections are a frequent complication of thalassemias and hemo-globinopathies and they can be fatal. The morbility and mortality rate for infections vary throughout the world depending on differences in the epidemiology of each infection and on the socio-economic level of each country and also vary depending on the preventive and therapeutic strategies adopted. In an Italian multicenter study^1^, infections were the second cause of death after heart failure in thalassemia. Similar results were reported in Greece^2^ and in Taiwan^3^, while in E-beta thalassemia patients in Thailand, infections are the primary cause of morbidity and mortality^4^.

Considering infections in sickle cell disease (SCD), the data are much more variable. In an analysis performed on 306 autopsies of SCD patients between 1929 and 1996, infections are the most common cause of death in all age groups (33–48%). The predominant anatomic site involved (72.6%) was the upper respiratory tract^5^. On the other hand, Darbari et al^6^, in 141 autopsies in SCD patients between 1976–2001, reported a lower mortality rate due to infections (18.4%) and infections were the fourth cause of death after pulmonary hypertension (PHT), the and renal failure. Both of these studies were conducted in USA. Perhaps the difference between these two reports reflects an improved surveillance of infectious complications. Bacterial infections are the main cause of death in Angolese SCD patients (40.1%)^7^. In France and England infections are the third cause of death and the rate is much lower (19%)^8^. A cohort study on children affected by SCD shows that the therapeutic strategy currently in use (transfusions, bone marrow transplantation, vaccinations and penicillin prophylaxis), decreased the global childhood mortality, in particular that which derived from infections, and it increased the mean age at the time of death^9^.

In this review we will compare and contrast the different mechanisms which predispose to infectious complications in thalassemia and in hemoglobinopathies, specifically SCD. We will distinguish between those aspects deriving from the disease itself and those which are essentially therapy related. Thereafter, we will examine only selected issues from the large amount of data on the clinical management of infectious diseases, trying to determine if there are infections to which these patients are naturally susceptible and others that are primarily due to treatment. Finally, the last point on which we will focus is how much some clinical aspects of these diseases (for example iron overload (IOL), and splenic absence (or hypofunction) influence the outcome of certain infection such as Acquired Immunodeficiency Syndrome (AIDS), hepatitis C virus (HCV) or bacterial infections.

## Etiology Of Risks Of Infections In Thalassemia And Hemoglobinopathies:

The susceptibility to infections in thalassemia and SCD arises both from a large spectrum of immunological abnormalities and from the exposure to infectious agents.

To simplify the complex scenario of immune system perturbations, four fundamental issues can be addressed: the disease itself, i.e. all those changes inherent to the pathological process which can interfere with the immune systems; IOL, transfusion therapy and the role of the spleen.

Transfusion and chelation therapies represent true progress in the management of these diseases. In fact, they dramatically ameliorated the prognosis of thalassemia and SCD, as epidemiological data clearly demonstrate^1^,^2^,^9^. Nevertheless, the benefits offered by allogenic blood transfusions (ABTs) come together with the disadvantages of the high transfusion burden in terms of direct exposure to infectious risks and, indirectly, transfusion related immunomodulation (TRIM) and IOL. Moreover, other therapeutic options (splenectomy, central venous catheters, bone marrow transplantation) or nutritional deficiency (zinc deficiency) contribute to the infectious risks.

### Immunological Abnormalities In Thalassemia And SCD:

Recently, the immunological abnormalities observed in thalassemic patients were reviewed and listed in two publications^10^,^11^. The immune alterations concern both the innate and the adaptive immune systems. The CD4/CD8 ratio is lower than normal, neutrophil and macrophage phagocytosis, neutrophil chemotaxis, natural killer (NK) function are compromised; C3 and C4 are reduced. High immuglobulins (Ig) were reported and B lymphocytes were found to be increased, activated with impaired differentiation. Table 1 summarizes the most important evidence in the literature (experimental or clinical), indicating, where noted, the relationship between the immune alteration and the ABTs or the IOL. There are few inconsistencies among the various reports.

The role of the disease itself in inducing immune abnormalities can be explained by pathophysiological mechanisms of the disease, as is reported in the literature.

The pathogenesis of thalassemia is based on ineffective erythropoiesis, hemolysis, and a tendency to increased iron absorption, inherent in the disease itself. For the first two reasons, the monocyte/macrophage compartment undergoes gross hyperplasia and is hyperactive in phagocytizing all defective erythroid precursors and erythrocytes^39^,^40^,^41^. This increased phagocytic activity very likely reduces the capacity of the phagocytic system to defend against pathogenic microorganisms. For the same reason, the pattern recognition receptors (PRR) are overwhelmed 28. Moreover, in a study conducted in a mouse model of β-thalassemia, susceptibility to infection by L. Monocytogenes and of S. Typhimurium was demonstrated as a result of low phagocytotic activity 13. The authors suggest that, in this model, the relationship of this alteration to IOL not caused by transfusions but results from the disease itself.

Finally, in clinical practice, it has been observed that severe anemia, itself, is a risk factor for bacterial infections in thalassemia, predominantly pneumonia^4^ ^43^. The current criteria for transfusion therapy recommend the maintenance of Hb level above 9 g/dl but in some countries with lower socio-economic levels, this optimal regimen is not assured. In these cases, anemia itself represents another risk factor for infections.

As far as SCD disease is concerned, its pathogenesis is quite different from thalassemia. Ineffective erythropoiesis does not play a central role as in thalassemia. HbS polymerization is the trigger, able to initiate the catastrophic chain of events responsible for chronic hemolytic anemia and for vaso-occlusive (VOC) crises. The latter may cause organ damage in all parts of the body and it accounts for the enormous clinical complexity of this disease. Much evidence is consistent with the existence of a chronic inflammatory state in SCD, exacerbated during the VOC episodes^44^,^45^ with participation of cells (neutrophils, macrophages platelets), cytokines and adhesion molecules. Many signs of high oxidative stress and decreased anti-oxidant defense are present^46^. Moreover, high interleukin-6 (IL-6) levels were observed in SCD^47^,^48^ in addition to interleukin-4 and interleukin-10^48^, ^49^. This cytokine elevation suppresses humoral and cell-mediated immune function, increasing infectious risks^49^,^50^. High values of soluble IL-2 receptors (sIL-2R), observed in a large number of SCD patients, were interpreted as the effect of continuous IL-6 stimulation^51^.

Regarding the cellular aspects of the immune system, monocytes are continously activated, as is demonstrated by the upregulation and the atypical expression of CD1^52^. Neutrophil dysfunction was considered a very important functional defect involved in the high susceptibility to infections^53^. For example, neutrophils from SCD patients show high expression of CD18, a molecule correlated with adhesive properties, and they respond, in vitro, to IL-8 with enhanced sensitivity^54^. This feature renders neutrophils important participants in the initiation of vaso-occlusion (VOCs) but they are thus less available for defense tasks.

In fact, VOC crises are responsible for further immune abnormalities which are present to a lesser degree or absent in the steady state of the disease 55. For example, phagocytic activity rises during VOCs 56. Neutrophil chemotaxis is normal or clearly reduced in the steady state of the disease but increases during VOC crises^57^. This hyperactivity of the monocyte/macrophage and neutrophil compartments is not committed to defending against pathogens but it contributes to VOCs. Moreover, it is a source of oxidative stress which impairs the immune response (see below).

As a further sign of inflammatory activation, the alternate (pathway of complement (AP50) is reduced for consumption in SCD patients and has a significant inverse correlation with the number of crises, while circulating immune complexes are elevated and they directly correlate with the number of complications of the disease 58.

The last factor to consider is that in SCD, VOCs themselves can predispose, locally, to the onset of infectious complications. Respiratory infections, frequently following the acute chest syndromes (ACSs), or osteomyelitis are examples of this mechanism^59^.

Another difference between thalassemic and SCD patients concerns splenic function: SCD patients undergo functional asplenia due to recurrent episodes of vaso-occlusion in this organ. Thus, the immunodeficiency observed in thalassemia after splenectomy is often naturally present even early in the life in SCD^60^. This state particularly favors infections by encapsulated bacteria^61^.

Finally, we mention that some immune alterations similar to those mentioned for thalassemia were also found in SCD: CD4 lymphocyte reduction and CD4/CD8 ratio reduction^55^, ^62^–^64^; natural killer lymphocyte reduced activity^64^; high serum immunoglobulin^65^, and elevated B lymphocytes^55^. On the other hand, the published data are less uniform and there are also some studies reporting the normality of these immunological features^66^,^67^.

### Risks Related To Iron Overload:

Hereditary hemocromatosis patients represent an ideal model to understand the effects of IOL on immunity. Indeed, many studies have demonstrated that immunological function is largely and negatively influenced by iron excess^68^. Many of the alterations observed in hereditary hemochromatosis were confirmed also in thalassemic patients (Table 1).

**Figure 1.**
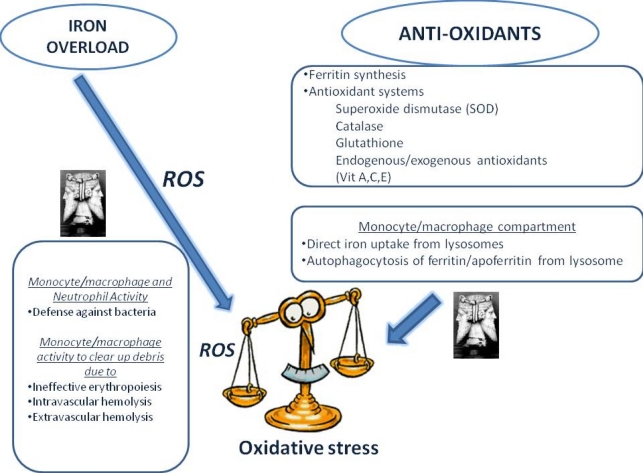
Risks related to iron overload.

To comment on the numerous data, we will outline only some specific aspects: for example the dual and opposing roles of the phagocytic system (monocyte/macrophages and neutrophils). IOL damage derives from a disequilibrium between iron oxidation (through the Fenton reaction) and the effectiveness and availability of those systems able to counteract oxidative stress. In this sense, in addition to the antioxidant systems, ferritin and the monocyte/macrophage compartment also participate in clearing up toxic iron. Indeed, lysosomes in these cells are able to endocytose both free iron and ferritin and this contributes toward protection from iron^68^ (Figure 1). Additional oxidative stress can destabilize the secondary lysosomes of the macrophage, and their protective role is lost. Moreover, phagocytosis of microorganisms, of dyserythropoietic precursors and of senescent or damaged red blood cells (intravascularly and/or extravascularly) causes oxidative stress 69 which compounds that deriving from IOL. Finally, IOL impairs phagocytosis^70^ and its negative effect on neutrophil functionhas been clearly demonstrated^70^,^71^. Phagocytic function is the center of a vicious cycle, acting as a double edged sword: protective against oxidative stress while also generating oxidative stress on the one hand, and on the other hand, having its own function impaired by the same oxidative stress (Figure 1).

Finally, the scanty detoxifying properties of lymphocyte are the reason for their numerous functional alterations related to IOL.

In addition, regarding IOL, SCD seems to be a different disease. Indeed non transfused SCD patients may present with iron deficiency (due to intravavascular hemolysis)^72^ and even in transfused patients, the organ damage due to iron overload is less severe^73^. Perhaps this difference derives from the significant contribution of inflammation to the pathogenesis of the disease, as recent studies evaluating the role of hepcidin in these diseases have led us to hypothesize^74^. A recent multicenter prospective study^75^ seems to support the influence of ABTs and IOL on the prevalence of infections requiring hospitalization, and, in general, on the rate of hospitalization, in SCD patients. Nevertheless, the data analysis shows a very complex scenario and the results suggest that this topic needs further studies to be clarified. Indeed, the transfused SCD are overall adult patients with more severe and advanced disease and, as the authors conclude, the differences observed may be, but not necessarily, attributable to ABTs and to IOL.

We conclude by mentioning that in patients who underwent hematopoietic stem cell transplantation, IOL severity is related to high infectious risk and it negatively influences the outcome of infections in this patient group^76^.

### Risks Related To Allogenic Blood Transfusions (ABTs):

The data regarding transfusion transmitted infection (TTIs) risks in patients with thalassemia and hemoglobinopathies does not differ from the evidence in the literature regarding multitransfused patients (MTPs) in general. Hepatitis C virus (HCV), Hepatitis B virus (HBV), Human Immunodeficiency virus (HIV) and Syphilis are the most common infection agents transmitted via transfusions and routine screening is performed for these agents throughout the entire world. Other agents are routinely screened for, in different countries, according to epidemiologic alerts but also commensurate with economic resources. In the USA, for example, screening for Human T-cell Lymphotropic virus (HTLV), West Nile virus (WNV), Trypanosoma cruzi and Cytomegalovirus (CMV) is also routinely performed on blood units and screening is performed for bacteria in platelet units^77^. Many other infectious agents are transfusion transmissible. The data in the literature demonstrated that some of these agents do not cause any clinical disease (GBV-C/HGV, SEN-V, TTV, HHV-8) while others represent a transfusional risk according to epidemiologic evidence. Thus, the risk of these agents can vary in different parts of the world. As summarized by Vanvakas et al 77 additional infectious agents which can be transmitted by transfusion include: Parvovirus B19, Dengue fever virus (DFV), Babesia microti, Plasmodia species, Leishmania, Brucella and Creutzfeldt-Jakob disease (vCJD) prions.

The prevention of HBV, HCV and HIV transfusion transmission represented a challenge for transfusion medicine. Two weapons play a fundamental role in the war against these viral agents. The primary preventive measure is the selection of appropriate eligibility criteria for blood donors; the second line of prevention includes testing the units to be transfused by various laboratory methods. Both tools have been and are always in continuous evolution. Health surveillance throughout the world, including rapid information about disease epidemiology and travel patterns of people, as well as the economic and political choices of each country and technological progress, have all contributed in the past and continue contributing to assure transfusion safety. Since the discovery of HBsAg in 1963, diagnostic accuracy has improved progressively. The introduction of Nuclear Amplification Tests (NAT) represented a milestone. A suitable example is transfusion transmitted HCV and HIV. Recently, the centralized data of the American Red Cross blood donor population were reviewed^78^ and the prevalence rates of disease marker positivity and the residual risk attributable to the window period were evaluated. A continuous statistically significant decrease (p<0.001) of prevalence rates for infectious disease markers among first-time donors was observed in the period between 1995 and 2001. Examining the data, the effect of the introduction of NAT testing is clear: the estimated risk of collecting blood during the infectious window period for HCV was 1:276,000 and 1:1,935,000 respectively with only antibody determination compared to NAT, respectively. Similarly, the risk for HIV was 1:1,468,000 and 1:2,135,000. The important role of the introduction of NAT is indirectly confirmed by the evidence that a less impressive reduction rate was recorded for HBV for which no relevant diagnostic improvements were achieved (1:205,000). Furthermore, another interesting approach to TTI evaluation is the application of mathematical models to calculate the residual risk of infection. The results obtained in the USA^79^ for HCV, HBV and HIV, are similar to those reported by Dodd et al. In England 80 and in Canada 81 the residual risk is substantially lower, in comparison to the USA, for HCV (1: 30 million and 1:13 million respectively) while for HIV only in Canada the residual risk is lower (1:7–8 million). Many clinical reports can be quoted to demonstrate the effect of the more advanced diagnostic tools adopted in transfusion field. For example, in Italy, a recent epidemiologic study of 708 multitransfused children, showed that HCV hepatitis, transmitted by transfusion, disappeared after 1992^82^. Furthermore, in another Italian study, performed retrospectively from 1990 until 2007, HCV-RNA negative thalassemic patients were significantly younger than positive patients (p<0.001)^83^. A survey of 399 patients with thalassemia and SCD in Turkey^84^ reported a prevalence of 0.75%, 4.5% and 0 of positivity to HBsAg, HCV and HIV antibodies respectively but the majority of this positivity (77.7%) was found in patients transfused before the introduction of second generation testing. The most recent data, although encouraging, suggest some considerations: different levels of blood safety are achieved among various countries. It derives that donor screening strategies can be ameliorated. Finally the problem of HCV and also HBV (we will expand on this below) is far from a complete resolution.

As far as the influence of ABTs on immune system is concerned, over 30 years ago, it was noted that patients who had received many ABTs prior to renal transplantation showed a better rate of allograft survival. This was the onset of a long and heated debate focused on understanding the immunomodulation induced by ABTs^85^–^87^. The debate initially began from the data of approximately 40 studies which indicated that surgical patients receiving perioperative ABTs have a higher risk of bacterial infections, demonstrating the link between multiple transfusions and infectious risk. Recently, Vamvakas and Blajchman 87 reviewed extensive evidence regarding this issue, summarizing the beneficial and deleterious effects of ABTs. TRIM could contribute to all immunological alterations listed above and it also reduces delayed-type hypersensitivy and it induces antiidiotypic and anticlonotypic antibody production. A central role in pathogenesis of TRIM is played by allogenic mononuclear cells, both for their presence and for the soluble substances they release during storage of blood components. Moreover, the soluble HL-A class I peptides that circulate free in allogenic plasma also contribute to the generation of TRIM. The similarity between donor WBC HLA antigens and those of the recipient is able to induce alloimmunization (if HLA-DR mismatch is high) or tolerance and immunosuppression (if the mismatch is for only one HLA-DR antigen). For these reasons, universal blood unit leukodepletion in the prestorage phase should be an important measure to prevent TRIM. Thalassemic patients represented an ideal setting to verify the usefulness of ABT leukodepletion. Although leukodepletion reduces non-hemolytic febrile reactions (NHFR)^88^–^90^ and anti-leukocyte antibodies and anti-platelet production^91^, ^92^ it does not modify substantially the immunologic alterations observed in thalassemic patients 92 Probably, their pathogenesis is very complex and TRIM represents only one of the numerous factors interfering with immunity.

### Risks Related To Splenectomy Or Functional Asplenia:

At the present time, as an effect of the hypertransfusion regimen, fewer thalassemic patients undergo splenectomy 93. However, when transfusional needs rise excessively, splenic enlargement, or hypersplenism and/or compressive damage occurs, splenectomy is indicated. We already outlined that SCD patients often present with functional asplenia early in life.

The spleen is very important for immunological surveillance. It is an important reservoir of immunocompetent lymphocytes^94^. In asplenia or functional hyposplenia, antibody production in response to new antigens, mediated by CD4 function, is impaired^95^. Efficient phagocytosis depends on splenic macrophages and on the production of many substances (opsonins, properdin, tufsin) which are reduced in asplenic organisms^96^, ^97^. Chemotaxis is also impaired 98. For all these reasons, when the spleen is absent or poorly functioning, sepsis can occur for any pathogen agent. However, encapsulated pathogens (Streptococcus pneumoniae, Haemophilus influenza type B, Escherichia coli, Neisseria menigitidis) are the most fearsome. Hansen et al^99^ reviewed the literature regarding overwhelming sepsis in subjects with surgical or functional asplenia. They compared the number of events of sepsis and fatal sepsis in recent reports to the same data obtained in 1973^100^. In 1973, sepsis occurred in 119 of 2796 cases (4.3%) and fatal sepsis occurred in 71 (2.5%). In the most recent series, sepsis occurred in 270 of 7872 cases (3.5%) and was fatal in 169 (2.1%) The percent reduction of sepsis from 1973 to most recent years was estimated -18 for sepsis and -16 for fatal sepsis. In both series, thalassemia patients have the highest frequency of sepsis and fatal sepsis. No comparison was possible for SCD because data before 1973 were lacking. The preventive strategy based on penicillin prophylaxis and vaccinations (see below) has been fundamental for this reduction of sepsis and fatal sepsis.

### Zinc deficiency:

The link between zinc deficiency and immunodeficiency is well known 101. Some reports, concerning SCD patients focus on this aspect and the beneficial role of zinc supplementation^102^,^103^.

## Selected Topics Regarding Clinical Aspects Of Infections InThalassemia And Hemoglobinopathies:

The amount of published data on the clinical aspects of infections in thalassemia and hemoglobinopathies is enormous and it is difficult to summarize it. In part, they are recently reviewed by Vento et al^11^. In the following section, we will focus on some specific aspects or new evidence arising from the literature, concluding by emphasizing the importance of preventive measures in splenectomized patients.

### Human Parvovirus B19:

Human parvovirus (HPV) B19 is a small, non enveloped, single stranded DNA virus with a terminal hairpin^104^. During replication, two proteins (VP1 and VP2) are produced but also in the absence of replication it can exert its toxic effects. After infection, a transient high titer viremia lasts one week; the HPV DNA disappears during the production of neutralizing antibodies (IgM for 6–8 weeks and afterwards, IgG). This protective reaction can be absent in immunocompromised patients leading to the persistence of viral DNA. The clinical course is characterized by a flu-like syndrome (fever, chills, headache, gastrointestinal discomfort, arthropathy and a typical slapped-cheek rash which, after two days also involves the arms and legs), sometimes complicated by a transient red cell aplasia (TRCA). In fact, HPV B19 it is also called erythrovirus because it has a high and almost specific tropism for erythroid progenitors inducing them to undergo apoptosis by the activation of the caspase pathway. In subjects with high erythroid turn over (such as those with congenital red cell defects) severe anemia with low reticulocyte counts may develop, requiring transfusion or an intensification of a previous transfusion regimen. Moreover, it is presumed that the virus can stay in the bone marrow for lifelong duration, although this point is not completely clarified and there is evidence that persistently infected blood donors can transmit the infection through transfusions 105, although the main route of transmission is always respiratory. For these reasons the course of HPV B19 infection in thalassemia and hemo-globinopathies can be quite different from that in a healthy subject.

A large epidemiological study of 633 children with SCD (older than 12 months) has been reported^106^. They were examined between November 1996 and December 2001. At the start of the study, 187 children (29.5%) had already contracted the disease (HPV B19 IgG+ and IgM-); their mean age was higher than that of serologically negative subjects (p<0.001) and fewer underwent chronic therapies (regular ransfusion or hydroxyurea-HU). The second cohort of patients (446; 70.4%) included those completely negative (IgG and IgM-) and those with a recent infection (IgG-, IgM+). The follow up of 372 children belonging to this group revealed important information: the rate of seroconversion; the features of seroconverted subjects, the prevalence of TRCA (severe or mild) and the variables related to the clinical course.

One hundred-ten children (29.5%) seroconverted during the follow up (incidence rate 11.3 for 100 patient-years; 95% confidence interval [CI] 8.2–14.4). It is very interesting that among them, fewer were receiving transfusions (7 out of 49; 14.3%; incidence rate 5.9 for 100 patients years, 95% CI 1–15) than those treated with hydroxyurea (9 out of 29; 31%) or not transfused (global incidence rate for non-transfused and HU groups: 11.9 per 100 patients years; 95% CI 7.6–16.2 p<0.06). Moreover, the only risk factor for seroconversion was having a sibling with a recent HPV B19 infection. These data can be important for what we will discuss later. SCD genotype, sex, age at the first serological test did not affect seroconversion.

Sixty-eight TRCA were observed during the study: 3 in the HPV B19 IgG positive group (1.6%) and 65 in the other (59%). The univariate analysis showed a strong association between acute HPV B19 infections with fever and acute splenic sequestration (ASS), while the multivariable analysis identified predisposing factors as ASS and painful episodes. Although the same evidence was not clear for acute chest syndrome (ACS), examining all children admitted with fever and pain, ACS was more common in those with HPV B19 infections. The only risk factor for TRCA was the high reticulocyte count before the infection. This study is rich in information and outlines many aspects of an infectious disease which has some peculiarities in SCD as compared to other diseases with high erythropoietic turnover. Nevertheless, an important debate is taking place in the literature as to whether transfusions are an important source of HPV B19. This hypothesis arises from the detection of HPV B19 DNA in asymptomatic blood donors. In the previous report^106^, treated children (transfusion or HU) seemed to have less seroconversion, perhaps because a lower proliferation rate of the erythroid compartment. Other reports coming from the transfusion medicine field^107^–^109^ support the evidence that transmission of HPV B19 through transfusion always plays a secondary role compared to respiratory transmission. As a result, there is currently no consensus regarding the application of preventive measures to blood donors, blood units or to patients.

### Yersinia Enterocolitica:

The well known problematic of Yersinia enterocolitica sepsis in thalassemia is another area in which some features of the disease combined with the side effects of therapy increase the risk of infection. In fact Yersinia infection is favored by IOL either related to the disease or to transfusions and it can be triggered by deferoxamine therapy 110, 111

## Transfusion Transmitted Infections (TTI)s:

In a manner analogous to the risks of infectious diseases, the course and the outcome of the most common TTIs in thalassemia and hemoglobinopathies are influenced by the pathogenic features of these diseases in terms of immunodysfunction and by IOL.

## HIV:

Human Immunodeficiency Virus (HIV) disease is a viral- related progressive immune depression that leads to depletion of CD4+ lymphocytes, and renders the individual at risk for many types of opportunistic infections^112^. As previously stated, a low CD4/CD8 ratio is one of the most frequent abnormalities in patients with thalassemias and hemoglobinopathies; thus, HIV disease is an example of negative interactions and bidirectional combination of the hematological with the infectious disease. Similarly, the substantial degree of immunodysfunction related to IOL would influence the outcome of these diseases. However, there are all too few studies dealing the clinical aspects of HIV infection in thalassemia and hemoglobinopahies.

Some years ago a large multicenter study was published^113^ which included 79 HIV positive thalassemia patients from various countries (Brazil, Italy, Greece, Spain, France, United Kingdom, Cyprus), the majority of whom were followed in Italy (71%) and Cyprus (16%). The mean age was low enough (12 ± 6.6 years) to presume a prevalent transfusion transmission of HIV infection. The progression to overt AIDS after seroconversion was estimated 1.4% after three years and 9% after five; no significant statistical association was found with age, sex, acute infection, or splenectomy. Two years later, the same investigator focused on the inverse relationship between the rate of progression of HIV and the dose of deferoxamine used: the rate of progression decreases as the mean daily dose of drug increases (p<0.02)^114^. In a further publication^115^ reporting the follow-up of the same patients, a multivariate Cox proportional hazard analysis demonstrated a direct relationship between disease progression and ferritin values. These studies, published at the beginning of the nineties, included some patients treated with zidovudine. In subsequent years until the present time, a large spectrum of therapeutic options are available for HIV infected patients: nucleoside analogues (NAs), non nucleoside reverse transcriptase inhibitors (NNRTIs), protease inhibitors (PIs), fusion inhibitors, CCR5 (receptor) inhibitors and integrase inhibitors^116^, which are used also in patients with thalassemia and hemoglobinopathies. Finally, we mention that the effect in vitro of iron chelators (deferoxamine, deferiprone, deferasirox) on HIV replication is an interesting area of experimental research^117^, ^118^.

## HCV:

Hepatitis C Virus still represents a fearsome disease, widespread worldwide: it is estimated that one hundred million people are infected throughout the world 119. It can have a mild presentation, not infrequently asymptomatic, in its acute phase and in a high percentage of cases, the initial infection goes unnoticed. However, the evolution rate to chronic disease of HCV hepatitis is high (at least 80% of acute cases) and the further evolution towards end-stage liver disease, cirrhosis, and hepatocellular carcinoma (HCC) are not infrequent^120^.

The influence of IOL on the outcome of HCV infection was the subject of debate both in nonthalassemic^121^,^122^ and thalassemic patients.

Di Marco et al^83^ reported that, in thalassemics, the severity of liver damage (i.e. the finding of fibrosis and histologic signs of cirrhosis) is clearly related to persistent HCV infection (HCV RNA positivity), predominantly for genotypes 1 and 4. In the same study, the data on the influence of IOL on liver damage in HCV RNA positive patients, although less impressive, are however suggestive. Many other authors focused their attention on the relationship between IOL and the outcome of HCV; although these studies may reflect some reporting bias, the results consistently demonstrate the presence of this negative link^123^–^128^. Much important evidence was obtained in patients who survived hemopoietic stem cell transplantation: serial liver biopsies, performed to evaluate histology and hepatic iron content, demonstrated that either HCV or IOL are independent risk factors for the progression of liver fibrosis and they have an additive effects^129^.

Since the 1990’s, the management of HCV has been characterized by remarkable improvements which initially began with the use of α-Interferon 2a (α-IFN). The first clinical results obtained with α-IFN were encouraging^130^, ^131^. α-IFN also showed long term efficacy^128^: 36.5 months (range 25–49 months). Syriopoulou^132^ reported a complete sustained response after 8 years of therapy in 45% of thalassemic patients. In the first of these two studies, upon multivariate analysis, the absence of cirrhosis, low iron content and infection with non 1b C virus type were independently associated with a complete sustained response. In the second study, younger patients, who were not splenectomized, with a shorter duration of the infection, were more likely to respond to therapy. α-IFN was used also in patients after bone marrow transplantation: it did not adverse engraftment and was demonstrated to be efficacious and safe^133^.

Thereafter, treatment options were enriched by the introduction of pegylated IFN (PegIFN) and ribavirin. There is currently an ongoing debate regarding the use of a combination of α-IFN (or Peg-IFN) plus ribavirin in the treatment of HCV in thalassemia. This option could be considered at least for patients infected by type 1b virus which results in a more severe disease and it is resistant to α-IFN as a single agent. On the other hand, it is well known that ribavirin is able to induce hemolysis and so in thalassemic patients the drug could increase the need for transfusions, thus worsening IOL. Although this is a definite possibility, preliminary experiences^134^–^136^ with this combination are positive in terms of efficacy on HCV infection. Inati et al^135^ reported a complete sustained response in 62% of patients using both drugs in comparison to 30% using IFN monotherapy (p=0.19). The patients required more transfusions but no worsening of IOL was observed. After the discontinuation of antiviral therapy, blood consumption returned to pre-therapy level. Other authors^134^, ^136^ reported similar results.

The last point concerns SCD patients; Teixera et al^137^, described the histopathologic features of SCD patients with or without HCV. This work has many limitations, as the authors state. Nevertheless, it gives interesting information: liver damage in SCD was present in subjects infected with HCV. In those not infected, the liver changes were mild and, despite IOL, little fibrosis was present. These observations are consistent with those made by Harmatz et al 138 and they imply that SCD differs from thalassemia in terms of the interaction between iron overload and HCV in SCD.

## HBV:

The strategies adopted in transfusion medicine as far as the widespread use of vaccination against HBV has reduced the prevalence of this hepatitis among multitransfused patients. Nevertheless, HBV hepatitis is still a serious public health problem. The reasons for this phenomenon are related to several factors. The routes of infection can be different (transfusion as compared to sexual or perinatal); the patients can be overt (HBsAg+) or occult (HBsAg – or anti HBc+/HBsAg-) carriers; and the virus can be reactivated in the setting of immunosuppression. Finally, the protection offered by vaccination is not absolute 139. How can the risks be managed? All transfused patients (who were vaccinated) or those with HBsAg+, must be tested annually for all HBV markers. The appearance of anti HBc positivity is a very important event which mandates careful clinical evaluation

HBV may present as an acute hepatitis with a wide range of manifestations, from mild disease, sometimes asymptomatic, to a severe one which, in some instances, can evolve to fulminant hepatic necrosis which is not uncommonly fatal 140. Apart from the acute phase, between 2 to 10% of patients evolve to chronic liver disease, and thereafter, end-stage liver disease, cirrhosis and hepatocellular carcinoma (HCC)^141^. The first line treatment, available for chronic HBV disease, is α-IFN. This drug should be used for one year. During this period the goal of therapy should be the complete clearance of HBV^142^, ^143^. Unfortunately, only 25%–40% of patients are noted to have a good response and the use of other antiviral drugs (adefovir, tenofovir, lamivudine, telbivudine, and entecavir) is often necessary^142^. Unfortunately, the major drawback of such therapies is that they are not “curative”, i.e. these drugs can reduce the viral replication, but they do not achieve complete viral clearance. Nonetheless, treatment is considered effective when liver fibrosis does not progress to cirrhosis^144^.

## Prevention Of Bacterial Infections In Splenectomized Patients:

The risk of invasive bacterial infection in splenectomized patients is well known. The data collected by Bisharat et al 145 supports this concept. They reviewed 28 studies amounting to 6,942 well-documented patients, 209 of whom developed invasive infection (3%). The incidence of infection was highest among patients with thalassemia major (8.2%), and sickle-cell anaemia (7.3%). Furthermore, the highest mortality rates were observed among patients with thalassaemia major (5.1%), and sickle-cell anaemia (4.8%). Both incidence and mortality were significantly higher in children than in adults. Streptococcus pneumoniae was responsible for the majority of the infections (66%), with a 55.3% mortality rate. It is followed for incidence by H. influenzae type b, Escherichia coli, and Neiserria meningitides^146^. Less common causative bacteria are Staphylococci, Streptococci, Pseudomonas, and Salmonella species^147^. The highest mortality rates were attributed to gram negative bacteria (62%), and Neisseria meningiditis (58.8%).

Thus the prevention and treatment of bacterial infections in splenectomized thalassemia and SCD patients is a life-saving intervention. Adamkiewicz et al^148^, reviewing the records of 1,247 children born after 1983, reported a clear beneficial effect of pneumococcal conjugate vaccine in the reduction of the incidence of invasive pneumococcal disease.

Some issues are of particular interest for clinical practice: the optimal timing of vaccine administration, the efficacy of various vaccination strategies, the duration of penicillin prophylaxis, and the role of partial splenectomy. Splenectomized and hyposplenic patients must receive routine vaccination, including both live attenuated and killed vaccines^149^, but they should also be immunized against Streptococcus pneumoniae, H. influenzae type b, and Neisseria meningitides^147^,^150^. In the case of elective splenectomy, vaccinations should be completed at least 2 weeks prior to the date of surgery.

However, vaccination does not completely protect against infection with encapsulated bacteria^151^ and prophylactic antibiotics have a role as well. In a prospective multicentre randomized study in pediatric SCD patients aged <3yrs, penicillin prophylaxis reduced the incidence of pneumococcal bacteremia by 84%. There are no prospective studies in different clinical settings, but in a retrospective observation^152^, the incidence of post-splenectomy sepsis (PSS) infection and mortality were reduced, by 47% and 88% respectively, after the introduction of penicillin prophylaxis. The patients had undergone splenectomy for different reasons, but the most relevant characteristic of the series is that 70% of the patients were immunized (54% out of them only against pnemococcus). Consequently, antibiotic prophylaxis is recommended for all children <5 years of age, regardless of immunization status, for all asplenic children <5yrs, for a duration of at least for 2 years following splenectomy, since most series demonstrate that 50% of PSS occurs within this period^153^. The debate about the duration of prophylaxis is still open and the emergence of penicillin-resistant pneumococci indicate that alternate therapy may be warranted.

Notwithstanding the risk of overtreatment, the potential catastrophic clinical course of bacterial sepsis in the splenectomized individual induces the physicians to start antibiotics at the first sign of infection. Patients should carry a medical alert card to improve the speed and appropriateness of treatment of postsplenectomy sepsis.

Subtotal splenectomy may reduce the risk of postsplenectomy sepsis^154^. Nevertheless, there are not, at the moment, specific recommendations for this procedure which has technical drawbacks in this population including regrowth of the spleen and the need for reoperation^155^.

Thus, also after a subtotal splenectomy, the guidelines mentioned above for total splenectomy should still be applied.

## Conclusions:

Thalassemia and SCD each have a different pathogenesis and this implies some differences in the risks factors for infectious complications. The strong inflammatory imprint and the frequent functional asplenia early in life in SCD are the most important, although not the only, differences between the two conditions. Moreover, although transfusions and bone marrow transplantation are important modalities to treat or cure both diseases, the additional problems arising from these procedures or from their adverse effects (for example IOL), have different implications. The knowledge of these differences is essential to efficiently target future research in experimental and clinical fields and also to define the best practical approach in the prevention and in the treatment of infectious diseases in these complex patients.

Although much progress has been made, infectious diseases still represent a major challenge in the efforts for assuring these patients enjoy a good quality of life and prolonged survival. The complexity of infectious complications, involving different regions of the body demonstrates that satisfactory cooperation among specialists in various disciplines (hematology, microbiology, immunology, hepatology), both in experimental and in clinical fields, is fundamental. Moreover, as a consequence of routine use of transfusions in these patients, transfusion medicine plays a central role. Ultimately, infectious diseases in thalassemia and hemoglobinopathies represent an example for which global surveillance, involving countries throughout the world, coupled with an open exchange of information are essential for achieving a high standard of patient care.

## Figures and Tables

**Table 1:** MECHANISMS OF IMMUNE DYSFUNCTION IN THALASSEMIA. The most important immune dysfunctions reported in thalassemia are shown. Those studies focusing on a specific pathogenic link between the immune alteration and iron overload (IOL) or allogenic blood transfusions (ABTs) are indicated. The data are predominantly clinical.

**IMMUNE ABNORMALITY**	**PATHOGENESIS**	**REFERENCES**
IMPAIRED MONOCYTE/MACROPHAGE ACTIVITY AGAINST PATHOGENS		Sternbach^12^ 1987
	IOL	Ampel^13^ 1989* Pittis^14^ 1994
	ABTs	Sternbach^12^ 1987
IMPAIRED NEUTROPHIL FUNCTION		Matzner^15^ 1993
	IOL	Bassaris^16^ 1982 Van Ashbeck^17^ 1984 Van Ashbeck^18^ 1984 Skoutelis^19^ 1984; Cantineaux^20^ 1987
	ABTs	Grady^21^ 1985 Sternbach^12^1987
DECREASED NK ACTIVITY		Dwyer^22^ 1987 Sen^23^ 1989 Ezer^24^ 2002
	IOL	Akbar^25^ 1986
	IO ABTs	A Akbar^25^ 1986
REDUCED ACTIVITY OF COMPLEMENT SYSTEM		Sihnlah^26^ 1981
CYTOKINE DYSFUNCTION		Wanachiwanawin 271999 Ozinsky^28^ 2000 Ozturk^29^ 2001
	ABTs	Lombardi^30^ 1994
T LYMPHOCYTE DYSFUNCTION REDUCED CD4 ELEVATED CD8 REDUCED CD4/CD8		Umiel 311984; Dwyer 221987 Khalifa^32^ 1988; Sen^23^ 1989 Dua^33^ 1993; Ezer^24^ 2002,
	TaABT	Kaplan 341984; Grady^35^ 1985 Pardalos^36^ 1987; Hodge^37^ 1999
B LYMPHOCYTE DYSFUNCTION		Umiel^31^ 1984; Dwyer^22^ 1987 Sen^23^ 1989; Speer^37^ 1990 Dua^33^ 1993;
	ABTs	H Hodge^36^ 1999
ELEVATED Ig PRODUCTION	ABTs	Akbar^38^ 1985 Dwyer^22^ 1987

* marks experimental or in vitro studies.
